# Fatal anaphylaxis to intravenous ondansetron: A case report

**DOI:** 10.1002/ccr3.4110

**Published:** 2021-05-05

**Authors:** Kalyan Sapkota, Roshan Bhagat

**Affiliations:** ^1^ Department of Medicine Bharatpur Hospital Chitwan Nepal; ^2^ ICU Department of Medicine Bharatpur Hospital Chitwan Nepal

**Keywords:** anaphylaxis, case report, fatal, ondansetron

## Abstract

The safety and efficacy of ondansetron has led to its wider clinical use and this could increase unusual serious adverse events. Therefore, we emphasize the need for cautious use of ondansetron and beware and prepare for unusual adverse events.

## Graphical abstract

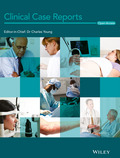

## 1 INTRODUCTION

Ondansetron is commonly used for nausea and vomiting. Serious adverse drug reactions are rare but not uncommon. We report a case who developed fatal anaphylaxis to intravenous ondansetron. This article emphasizes the need for cautious use of drugs and importance of recognition and treatment of this serious adverse event.

Ondansetron is a selective serotonin 5‐HT3 receptor antagonist, which is widely used in the prevention and treatment of chemotherapy‐induced nausea and vomiting. The most commonly reported side effects (occurring in more than 10% of adults) include headache, fatigue, malaise, and constipation. Serious side effects include QT prolongation and severe allergic reaction. Hypersensitivity reactions to Ondansetron are rare but have been reported.^1^, ^2^, ^3^, ^4^ Ondansetron can cause anaphylaxis, so one should be careful while using the drug.

## 2 CASE PRESENTATION

An 82‐year‐old woman presented to the Emergency Room (ER) at 0430 hours with complaints of acute onset of vertigo and multiple episodes of nonprojectile, nonbilious vomiting. She also complained of generalized weakness and dry mouth. Her past history was unremarkable except for the use of calcium supplementation and aceclofenac for pain due to osteoarthritis. On examination, she was afebrile, had pulse rate of 96/min, and blood pressure was 90/60 mm Hg. Patient was thin built, alert but slightly ill‐looking with dry tongue. Systemic examination was unremarkable.

She was treated with single 4 mg dose of Ondansetron (brand name “Tilset”) intravenously. Immediately following the medication, she became restless, developed respiratory distress, and quickly progressed to falling oxygen saturation, drop in blood pressure from 90/60 mm Hg to 70/40 mm Hg, and decreasing level of consciousness. Patient was cyanosed, and diffuse wheeze heard on chest auscultation. However, there were no urticaria, rashes, angioedema, or pruritus.

An allergic reaction to ondansetron was suspected, immediate resuscitation was started and 0.5 mg epinephrine (1 mg/1 mL) was given intramuscularly. Volume expansion with normal saline was initiated and bolus of IV Hydrocortisone and IV Pheniramine were administered. There was no improvement seen clinically and hemodynamically. Because of deteriorating condition as evidence by rapidly falling oxygen saturation, unstable hemodynamic parameters, and falling level of consciousness, airway was secured with endotracheal intubation. Bag and mask ventilation with 100 percent oxygen started and continued. Continuous noninvasive hemodynamic monitoring and pulse oximetry monitoring were performed throughout the period. As there was no response and no sign of improvement, IM Epinephrine 0.3 mg repeated every 3 minutes^5^, ^6^; however, the hypotension persisted and continuous infusion of adrenaline was started. With all these resuscitation attempts, SPO_2_ displayed on monitor was 54%. ECG monitoring showed bradycardia with heart rate of 30 bpm. Further drop in blood pressure was noted and was unrecordable immediately which quickly progressed to cardiac arrest. High‐quality chest compression was started and advanced cardiac life support was continued. However; return of spontaneous circulation could not be achieved and the patient died after 30 minutes of resuscitation due to refractory cardiorespiratory arrest.

She had no history of ondansetron exposure, drug, or food allergies. As no other drugs were administered and onset of signs and symptoms developed immediately after administration of ondansetron, it is believed that this reaction was most probably caused by ondansetron.^7^ Using Naranjo adverse drug reaction probability scale, the score was 5, the relationship between the drug and the event was categorized as “probable.”^8^

## 3 DISCUSSION

Selective 5‐HT3 receptor antagonists such as ondansetron, granisetron, dolasetron, and palonosetron are widely used for their antiemetic properties in cancer chemotherapy. They are generally associated with a wide safety margin; however, there are some reports of life‐threatening adverse events such as anaphylaxis and tachyarrhythmias. Although this drug is well‐tolerated, there have been multiple reports of adverse reactions associated with various clinical manifestations of anaphylaxis.^2^ Anaphylaxis is an acute, potentially fatal, multiorgan system reaction caused by the release of chemical mediators from mast cells and basophils. Anaphylaxis due to ondansetron could be due to immune‐mediated or nonimmune mediated. Exposure to ondansetron was unknown in our patient and, therefore, the exact mechanism for anaphylaxis could not be identified.

Treatment of signs and symptoms of anaphylaxis is epinephrine with cardiovascular and respiratory support. But, due to rapid onset and severe presentation in our case, even with aggressive treatment and all the efforts of providing cardiorespiratory support, patient could not be survived. Ondansetron was considered to be the event‐producing drug because respiratory and hemodynamic manifestation occurred immediately after injection and no other medications were given at that time. Because of the rapid onset of clinical event and life‐threatening condition, blood samples could not be obtained in our patient and skin testing could not be done.

Anaphylaxis to ondansetron is a rare event, but the easy availability of ondansetron has promoted the widespread off‐label use of these drugs in conditions other than clinically indicated in Nepal. Though rare but serious adverse events could occur with injudicious use of these agents, and simple treatment for the minor ailments could be life‐threatening. Our case report emphasizes the judicious use of ondansetron so as to avoid and reduce similar untoward events and we need to be cautious while using this drug and be aware when using it in out‐of‐hospital set‐up.

## CONFLICT OF INTEREST

None.

## AUTHOR CONTRIBUTIONS

KS: involved in the writing, revisions, and final review of the manuscript. RB: involved in the writing, revisions, and final review of the manuscript.

## ETHICAL APPROVAL

Written‐informed consent was obtained from the patient's legal guardian for publication of any detail that might identify an individual.

## Data Availability

The data that support the findings of this study are available on request from the corresponding author.
